# Brevetoxin PbTx2 Modulates Oxidative Stress and Inflammatory Response in an In Vitro Human Immune Cell Line

**DOI:** 10.3390/toxics14030238

**Published:** 2026-03-10

**Authors:** Ambbar Aballay-González, Claudia Melo, Alejandra Rivera, Miquel Martorell, Viviana Ulloa, Juan José Gallardo-Rodriguez, Allisson Astuya-Villalón

**Affiliations:** 1 Centro de Investigación Oceanográfica COPAS Sur-Austral, Universidad de Concepción, Barrio Universitario s/n, Concepción 4070386, Chile; amaballay@udec.cl (A.A.-G.); claumelo.astorga@gmail.com (C.M.); alejandrarivera@udec.cl (A.R.); 2 Laboratorio de Biotoxinas de la Universidad de Concepción (LBTx-UdeC), Departamento de Oceanografía, Facultad de Ciencias Naturales y Oceanografía, Universidad de Concepción, Barrio Universitario s/n, Concepción 4070386, Chile; viv.ulloa@gmail.com; 3 Departamento de Nutrición y Dietética, Facultad de Farmacia, Universidad de Concepción, Concepción 4070386, Chile; mmartorell@udec.cl; 4 Centro de Vida Saludable, Universidad de Concepción, Concepción 4070386, Chile; 5 Department of Chemical Engineering, Research Centre CIAIMBITAL, University of Almería, 04120 Almería, Spain; jgr285@ual.es; 6 Centro de Biotecnología, Universidad de Concepción, Concepción 4070386, Chile

**Keywords:** cell-based assay, brevetoxin-2, harmful algal bloom, ROS production, human monocytic cell line, oxidative stress, inflammation

## Abstract

Brevetoxins (PbTx) exert detrimental effects on marine organisms and humans, mainly through alterations in immune cell function. This study evaluated the immunotoxic potential of sublethal concentrations of PbTx-2 using the THP-1 human monocyte cell line as an in vitro model. Cell viability assessed by the MTT assay revealed an IC_50_ of 8.99 µM at 24 h, while exposures to 2.8 and 5.6 µM for 4 and 8 h did not affect viability. Immune and oxidative responses were examined through antioxidant activity and transcript expression by qPCR. PbTx-2 exposure altered the expression of catalase, glutathione reductase, interleukin *IL-8*, *IL-1β*, and *TNF-α*. Although reactive oxygen species (ROS) levels remained unchanged, catalase activity and Cu/Zn-superoxide dismutase activity decreased after 8 h. These results indicate that PbTx-2 modulates redox and inflammatory pathways in THP-1 cells, even under non-cytotoxic conditions. The observed sublethal effects suggest potential immunomodulatory consequences of brevetoxin exposure. More studies are needed to determine whether chronic low-level exposure to brevetoxins could contribute to immune dysfunction or inflammatory pathologies in humans and marine mammals.

## 1. Introduction

Neurotoxins such as brevetoxins (PbTxs) are produced during microalgal blooms of the Raphydophiceae class, such as *Karenia brevis* and *Chatonella* spp. [1,2]. Currently, a wide range of BTXs have been identified [3], which are produced naturally and classified as A or B according to their type of polyether structure. The main type-A PbTxs are PbTx-1 and PbTx-7, whereas the main type-B PbTxs are PbTx-2, PbTx-3, and PbTx-9 [4]. PbTxs are lipid-soluble cyclic polyether compounds known to activate voltage-sensitive sodium channels in mammals through a specific interaction with site 5 of the alpha subunit [5]. PbTxs cause an excitatory effect since their binding to the sodium channels inducing a leftward (more negative) shift in the voltage dependence of activation and slowing channel inactivation [6], which leads to an increase in the influx of sodium ions, altering the properties of the membrane of excitable cells, such as neurons, cardiac cells, and muscle tissue [5,7]. In humans, PbTxs cause neurotoxic shellfish poisoning via inhalation or ingestion. The naturally apolar transference and accumulation of these toxins are caused by the consumption of contaminated shellfish and fish, and are capable of easily binding to muscle and tissue of different organs [1,8,9]. The lethal doses of PbTxs are in the range of a nanomolar concentration [10,11]. Consumption of these toxins produces a combination of different gastrointestinal and neurologic symptoms [1,12,13,14]. Additionally, when cells of species that produce PbTxs are lysed, they can be aerosolised; in that case, they can cause symptoms such as dry cough, itchiness in the eyes and nose, asthma attacks, and worsening of chronic obstructive pulmonary disease [15,16,17,18,19].

The molecular/cell sites where the PbTxs are held in the tissue are unknown, although some possibilities include tissue lymphocytes and macrophages [20]. In addition, PbTxs can be associated with lipoproteins and be carried through the circulatory system [21]. Toxicokinetic studies in rats using radiolabeled PbTx-3 have shown detectable levels of toxin in the blood after administering it intravenously, intratracheally, and orally [22,23]. In addition, PbTxs polar metabolites can be detected in rat blood for up to 14 days after intraperitoneal injection [24]. Some studies have examined in vitro PbTx exposure on immune mammal cell lines, and it has been shown to affect growth, as well as proliferation [25,26]. The leukemic T cell line (Jurkat) showed a decrease in cell viability and proliferation, and an increase in apoptosis, as examined with the activity of caspase-3 when exposed to different concentrations of PbTxs [25]. Aberrant cell division was observed in murine myeloma SP2/O cells, associated with the accumulation of PbTxs in the cell nucleus [26].

Several studies have indicated that PbTx exposure produces oxidative stress and an inflammatory response [25,27,28,29]. In the plasma of manatees rescued after exposure to a red tide, the activity of superoxide dismutase (SOD) was significantly higher compared to either pre-release or non-exposed animals [27]. In human monocytic cells (U-937), PbTx-2 decreased intracellular glutathione [30]. In mast cells derived from the bone marrow of mice, PbTx-2 triggered degranulation and increased levels of interleukin (IL)-6 protein levels [28]. In a cell line of alveolar macrophages (MH-S), PbTx-2 caused an increase in phagocytosis and the release of IL-2, IL-4, and tumor necrosis factor (TNF-α) [29].

PbTxs are not currently regulated toxins in many countries, although the frequencies of harmful algal blooms (HABs) associated with Raphydophiceae are increasing, and the effects of sublethal exposures of these toxins on human health are unknown. Bioaccumulation of these toxins at non-lethal concentrations in sea environments can pose a threat to human health through the consumption of contaminated seafood within the food chain. The aim of this study was to examine the in vitro effects of PbTx-2 on the cell viability, oxidative stress, and gene expression of proinflammatory cytokines and antioxidant enzymes in a monocytic human leukemia (THP-1) cell line. Brevetoxin-2 (PbTx-2) was selected as the model congener for this study because it is the “parent” or prototype toxin among the brevetoxin B family; it is considered more abundant and exhibits high bioactivity [24,31,32,33]. The immunotoxic potential of PbTx-2 remains poorly understood; therefore, investigating the oxidative and inflammatory responses induced by PbTx-2 in immune cells can provide valuable information on its role in systemic toxicity and immunopathology associated with harmful algal bloom exposure.

## 2. Materials and Methods

### 2.1. Cell Culture

The THP-1 cell line (ATCC, Manassas, VA, USA) was obtained from Dr. Felipe Zuñiga and was maintained in RPMI 1640 (GIBCO, Thermo Fisher Scientific, Waltham, MA, USA) medium supplemented with 10% fetal bovine serum, 0.025 mM glucose, 1 mM sodium pyruvate, 2 mM L-glutamine, 100 IU mL^−1^ penicillin-streptomycin, and 2.5 µg mL^−1^ amphotericin B, and maintained at 37 °C in a humidified 5% CO_2_ atmosphere. For cell viability assay and the determination of intracellular ROS, THP-1 cells were seeded in 96-well plates (100 µL/well) at 2 × 10^5^ cells per well in RPMI 1640 medium supplemented with 10% of fetal bovine serum. Cells were exposed to the indicated PbTx-2 concentrations for 4, 8, or 24 h in a final volume of 110 μL. PbTx-2 dilutions were prepared by adding 10 µL of PbTx-2 solution to each well, ensuring that the final methanol (vehicle) concentration did not exceed 1% (*v*/*v*). The selection of these concentrations was based on previously reported IC_50_ values for immune cell lines and range-finding studies to identify mechanistic thresholds [25,32]. Although these levels exceed average environmental water concentrations (often in the µg L^−1^ range), they were used to facilitate detection of acute cellular stress and molecular initiating events within a short-term (24 h) in vitro exposure window, compensating for the lack of chronic systemic exposure and bioaccumulation present in whole organisms [34]. PbTx-2 was obtained from Sigma-Aldrich (Merck, Darmstadt, Germany). The toxin was supplied in an amount of 100 µg dissolved in 1 mL of methanol obtained from Sigma-Aldrich (Merck, Darmstadt, Germany); then it was aliquoted and stored at −20 °C until use. Control cells correspond to those treated with the vehicle (1% methanol), which exhibited approximately 100% viability. The same control was used for all measurements.

### 2.2. Half-Maximum Inhibitory Concentration and Cell Viability Assay

The half-maximum inhibitory concentration (IC_50_) was determined by treating THP-1 cells for 24 h with PbTx-2 at final concentrations ranging from 0.5 to 10 µg mL^−1^. Cell viability was assessed using the MTT assay (3-(4,5-dimethylthiazol-2-yl)-2,5-diphenyltetrazolium bromide; Life Technologies, Carlsbad, CA, USA). After exposure to PbTx-2, cells were centrifuged at 179× *g* for 10 min and incubated with 100 µL of RPMI-1640-supplemented medium containing 0.6 mg mL^−1^ of MTT for 4 h at 37 °C. Following incubation, the samples were centrifuged again at 179× *g* for 10 min, the resulting formazan crystals were dissolved with 100 µL of dimethyl sulfoxide, and absorbance was measured at 570 nm (Synergy H1Microplate Reader, Biotek, Winooski, VT, USA). The data is presented in percentages of living cells relative to the control (~100% viability). The experiments were performed in triplicate.

### 2.3. Intracellular ROS Production

For intracellular ROS determination, THP-1 cells were exposed to PbTx-2 at 2.5 and 5.0 µg mL^−1^ for 4 and 8 h. After exposure, intracellular ROS levels were measured using 2′,7′-dichlorofluorescein diacetate (H_2_DCFDA, Calbiochem, San Diego, CA, USA). Cells were incubated with 100 µL of phosphate-buffered saline (PBS, pH 7.4) containing 20 µM H_2_DCFDA at 37 °C for 45 min. Fluorescence (Ex, 480 nm; Em, 530 nm) was measured for 2 h using a Synergy H1 microplate reader (Biotek, Winooski, VT, USA). Experiments were performed in triplicate and included vehicle controls (1% methanol solvent) and 1 µg mL^−1^ Lipopolysaccharides (LPSs) (Sigma-Aldrich, St. Louis, MO, USA) as a positive control to verify the functional competency of THP-1 cells and the reliability of the ROS detection assay under standard inflammatory stimulation. Fluorescence data were expressed as relative fluorescence units (RFUs) relative to the control group.

### 2.4. Enzyme Activities

THP-1 cells were seeded in 12-well plates at a density of 1 × 10^6^ cells per well in RPMI 1640 medium supplemented with 10% fetal bovine serum and incubated for 4 and 8 h with PbTx-2 at concentrations of 2.5 and 5.0 µg mL^−1^. Lipopolysaccharide (LPS, 1 µg mL^−1^) was used as a positive control. Cells were then collected by centrifugation at 179× *g* for 10 min and washed twice with PBS pH 7.4. Finally, the cells were lysed in 1 mL HPLC-grade water and centrifuged at 10,000× *g* for 15 min at 4 °C. The supernatants were collected and stored at −80 °C until further enzymatic analyses. Catalase activity was measured by the spectrophotometric method of Aebi, based on hydrogen peroxide (H_2_O_2_) decomposition, hydrogen peroxide was obtained from Sigma-Aldrich (Merck, Darmstadt, Germany) [35]. Superoxide dismutase (SOD) activity was determined using an adapted method of McCord and Fridovich [36]. Enzyme activities were expressed as U mL^−1^ (µmol min^−1^ mL^−1^).

### 2.5. Gene Expression

For the qPCR assay, THP-1 cells were seeded at a density of 3 × 10^6^ cells per well in sterile 6-well culture plates and incubated for 4 and 8 h with PbTx-2 at concentrations of 2.5 and 5.0 µg mL^−1^. Experiments were performed in triplicate. Total RNA was extracted from THP-1 cells using TRIzol Reagent (Invitrogen, Carlsbad, CA, USA) according to the manufacturer’s instructions. RNA samples were treated with DNase I (Thermo Scientific, Waltham, MA, USA) and subsequently used for cDNA synthesis with the RevertAid H Minus First Strand cDNA Synthesis Kit (Thermo Scientific, Waltham, MA, USA) following the manufacturer’s protocol. RNA integrity was assessed by agarose gel electrophoresis in MOPS buffer. Primers were designed for the following genes: IL-1β (F: 5′-CTG TAC CTG TCC TGC GTG TT-3′; R: 5′-AGA CGG GCA TGT TTT CTG CT-3′), IL-8 (F: 5′-CTC CAA ACC TTT CCA CCC CA-3′; R: 5′-TTC TCC ACA ACC CTC TGC AC-3′); TNF-α (F: 5′-CTT CTC GAA CCC CGA GTG AC-3′; R: 5′-ATG AGG TAC AGG CCC TCT GA-3′); GRd (F: 5′-CAC ACC CAA GTC CCC TGC ATA T-3′; R: 5′-TCA CGC AGT TAC CAA AAG GAA-3′); GPx (F: 5′-TTC CCG TGC AAC CAG TTT G-3′; R: 5′-TTC ACC TCG CAC TTC TCG AA-3′); Cu/Zn-SOD (F: 5′-TCA GGA GAC CAT TGC ATC ATT-3′; R: 5′-CGC TTT CCT GTC TTT GTA CTT TCT TC-3′); catalase (F: 5′-TCC CCA TTT GCA TTA ACC AG-3′; R: 5′-TTT GGC TAC TTT GAG GTC AC-3′) and Eukaryotic translation initiation factor 2B, sub-unit 2 (EIF2B2) (F: 5′-TCC ACC CCA CTC ATC GTC TG-3′; R: 5′-TGG CAG GAC TTC TTC AGG AGC-3′). Amplification efficiency for each primer pair was evaluated and optimized using standard curves generated from serial cDNA dilutions, and only primer pairs exhibiting amplification efficiencies close to 100% were included in the analysis. The qPCR assays were performed using Maxima SYBR Green qPCR Master Mix (Thermo Scientific, Waltham, MA, USA) on an EcoTM Real-Time PCR System (Illumina, San Diego, CA, USA). Relative mRNA expression levels of the analyzed genes were determined using the comparative 2^−ΔΔCt^ method [37], which expresses changes in mRNA levels relative to the untreated control and normalized to the EIF2B2 reference gene.

### 2.6. Statistical Analysis

Statistical analyses were performed using Statistica software version 10.0 (StatSoft Inc., Tulsa, AZ, USA). Data were assessed for normality and homogeneity of variance using the Shapiro–Wilk test and the Levene test, respectively. The results were analyzed by factorial analysis of variance (ANOVA), followed by Tukey’s honestly significant difference (HSD) post hoc test. A *p*-value < 0.05 was considered statistically significant.

## 3. Results

### 3.1. Determination of IC_50_ and Cell Viability Assay

**Figure 1:**
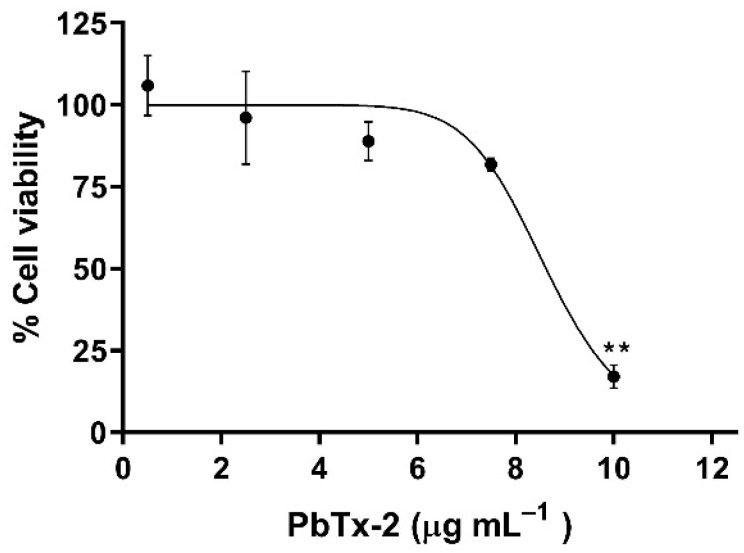
Dose–response curve for PbTx-2 over 24 h in THP-1 cells. Results are expressed as the percentage of cell viability relative to the untreated control with vehicle (mean ± standard deviation). Asterisks indicate statistically significant differences compared with the control, as determined by Tukey’s test; (**) *p* ≤ 0.01.

**Figure 2:**
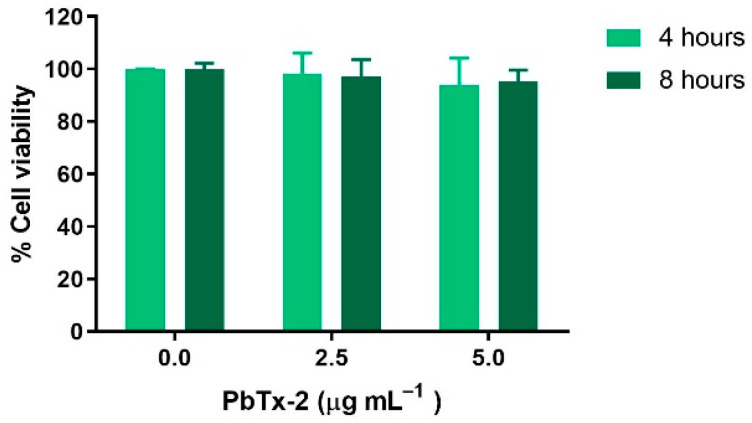
Viability of THP-1 cells exposed to 2.5 and 5.0 µg mL^−1^ of PbTx-2 for 4 and 8 h. Results are expressed as mean ± standard deviation.

The PbTx-2 dose–response curve for 24 h in THP-1 cells showed no significant differences between the control (no PbTx-2 exposure) and cells treated with 0.5–7.5 μg mL^−1^ (0.6–8.4 µM) PbTx-2, although cell viability decreased to approximately 80% at 7.5 μg mL^−1^ (8.4 µM) (Figure 1). The IC_50_ value was 8.047 µg mL^−1^ (8.99 µM). Treatment with the highest concentration tested (10 µg mL^−1^ or 11.2 µM) resulted in a statistically significant decrease in viability to 17%. Subsequent experiments were performed using concentrations below the determined IC_50_ to ensure sublethal conditions. Cells treated for 4 and 8 h with 2.5 and 5.0 µg mL^−1^ (2.8 and 5.6 µM) PbTx-2 did not show significant differences in viability compared to the control group (Figure 2).

### 3.2. Effect of PbTx-2 on ROS Production and Antioxidant Enzyme Activities

**Figure 3:**
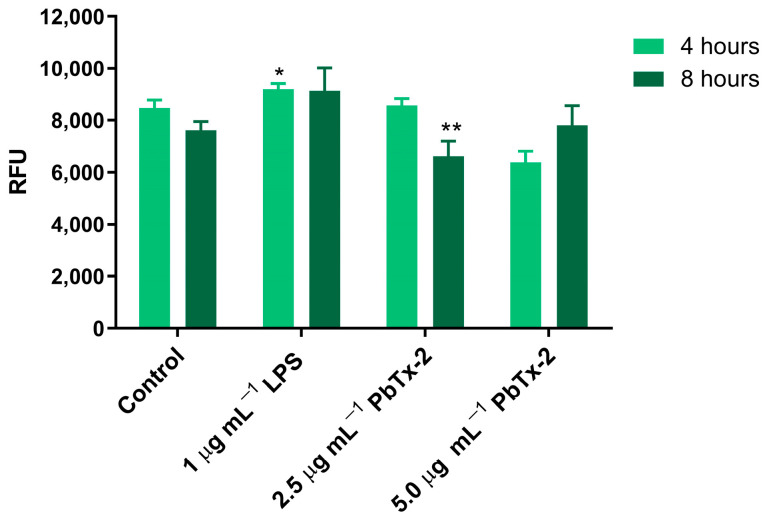
Reactive oxygen species (ROS) production in THP-1 cells exposed to 2.5 or 5.0 µg mL^−1^ PbTx-2 for 4 and 8 h. Fluorescence is expressed in relative fluorescence units (RFU). H_2_DCFDA was used as the fluorescent probe. Results are presented as mean ± standard deviation. Asterisks indicate statistically significant differences compared to the control group, as determined by Tukey’s test; (*) *p* ≤ 0.05, (**) *p* ≤ 0.01.

Exposure of THP-1 cells to 2.5 and 5.0 µg mL^−1^ PbTx-2 for 4 h did not cause any change in ROS production compared to the control group (Figure 3). Lipopolysaccharide (LPS, 1 µg mL^−1^) was used as a positive control, and a statistically significant increase in ROS was observed after 4 h of LPS treatment. After 8 h of exposure at 2.5 µg mL^−1^ PbTx-2, a significant decrease in intracellular ROS production was observed compared to the control group.

**Figure 4:**
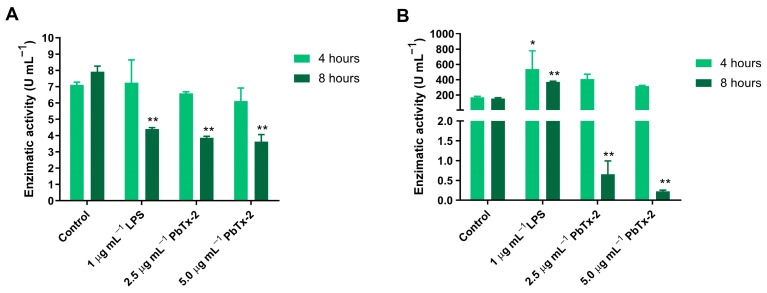
Enzyme activities in THP-1 cells after 4 and 8 h of exposure to 2.5 µg mL^−1^ and 5.0 µg mL^−1^ PbTx-2. (**A**) Catalase activity. (**B**) Cu/Zn-superoxide dismutase (SOD) activity. Values are expressed as the mean ± SD of each condition performed in duplicate. Asterisks indicate statistically significant differences compared with the control group, as determined by Tukey’s test; (*) *p* ≤ 0.05 and (**) *p* ≤ 0.01.

Figure 4 shows the enzyme activities of catalase (Figure 4A) and superoxide dismutase (SOD, Figure 4B) after 4 and 8 h of exposure to 2.5 and 5.0 µg mL^−1^ PbTx-2, with 1 µg mL^−1^ LPS as a positive control. For catalase, exposure to PbTx-2 for 8 h resulted in approximately a 50% decrease in enzyme activity for all treated conditions, except for the control group. For SOD, 8 h of PbTx-2 exposure caused a significant 32-fold decrease in enzyme activity compared with the control group.

### 3.3. Effects of PbTx-2 on Gene Expression

**Figure 5:**
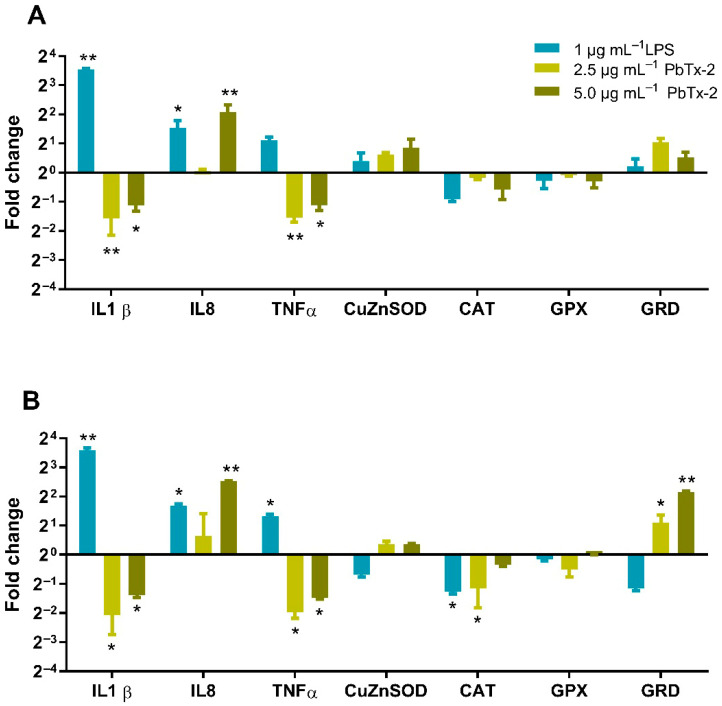
Fold change of pro-inflammatory cytokines and oxidative stress genes in THP-1 cells stimulated for 4 h (**A**) and 8 h (**B**) of exposure to 2.5 and 5.0 µg mL^−1^ PbTx-2. Gene expression was calculated using the log_2_[2^−ΔΔCq^] method, normalized to the reference gene *EIF2B2*, and expressed relative to the untreated control, which was set to 1 (Control = 1; 2^0^ = 1). Values > 1 indicate positive regulation, while values <1 indicate negative regulation. Asterisks indicate statistically significant differences compared with the control, as determined by Tukey’s test; (*) *p* ≤ 0.05 and (**) *p* ≤ 0.01.

The gene expression of the pro-inflammatory cytokines *IL-1β* and *TNF-α* showed similar profiles, with a decrease in transcriptional level upon exposure to 2.5 and 5.0 μg mL^−1^ PbTx-2 for 4 and 8 h (Figure 5). *IL-8* was upregulated, with expression increasing after 8 h of exposure to 2.5 and 5.0 μg mL^−1^ PbTx-2. Antioxidant enzyme genes, catalase and glutathione peroxidase (*GPx*) were downregulated following 4–8 h of exposure. In contrast, glutathione reductase (*GRd*) transcript levels increased after 4 and 8 h of treatment with 2.5 and 5.0 μg mL^−1^ PbTx-2. Regarding gene expression, while *Cu/Zn-SOD* mRNA levels remained relatively stable under the tested conditions, a significant decrease in catalase mRNA levels and a concomitant increase in *GRd* expression were observed at 8 h. These results suggest that the transcriptional antioxidant response to PbTx-2 in THP-1 cells is primarily driven by the glutathione cycle and peroxide detoxification pathways rather than superoxide dismutation at these specific time points. Notably, catalase expression was significantly downregulated after 8 h of treatment with 5.0 μg mL^−1^ PbTx-2.

## 4. Discussion

The mechanism of action of PbTxs on immune cells remains poorly understood; however, the available evidence suggests that these toxins modulate both innate and adaptive immune responses [38]. Several molecular and cellular mechanisms have been proposed, including the inhibition of cathepsin active sites [39], induction of apoptosis [20,40,41], release of inflammatory mediators [29] and effects on the cellular cycle [26]. In the present study, PbTx-2 significantly reduced cell viability only at the highest tested concentration (10 µg mL^−1^). However, Walsh et al. (2008) [25] reported that, in Jurkat E-6 cells, cell viability decreased significantly after 24 h of treatment with 2.5 µg mL^−1^ PbTx-2, and metabolic activity was reduced to 50% at 1.25 µg mL^−1^ (~1.4 µM). It should be noted that different cell lines were used in these studies, and cell types likely exhibit differential responses to PbTx-2 treatment. Indeed, several studies in immune cells indicate that the IC_50_ value for PbTx-2 ranges from 1 nM to 10 µM [28,32,33,41,42]. This wide range clearly illustrates the considerable variability in susceptibility among different cell types to PbTxs (e.g., Type-1: PbTx-1, -7 and Type-2: PbTx-2, -3, -6). For example for PbTx-2, the IC_50_ value has been reported as 5.6 µM in Jurkat E6-1 cells [32] and 0.18 µM in CHO-K1-BH4 cells [42]. McCall et al. 2022 [41] determined an IC_50_ value of 2.63 µM for THP-1 cells using the XTT cytotoxicity assay. The IC_50_ value determined in this study for THP-1 cells was 8.99 µM (8.047 µg mL^−1^ PbTx-2), which lies at the upper end of the established range and is comparable to values reported for other cell lines (e.g., Jurkat). The IC_50_ of 8.99 µM found in this study for THP-1 cells is higher than the values reported for lymphoid lineages. Murrell and Gibson (2010) [43] observed that T-lymphocyte cell lines (Jurkat) were more sensitive to PbTx-2, exhibiting rapid loss of membrane integrity and mitochondrial potential. However, unlike the fluctuations in SOD and catalase enzymatic activity observed in THP-1 cells, these T-cells did not display a significant oxidative burst. These findings suggest that the mechanisms of cell death may differ between cell types, with lymphocytes being more susceptible to direct ion channel disruption, whereas monocytic cells such as THP-1 involve more complex oxidative pathways.

Excessive exposure to environmental toxins, including PbTxs, can lead to increased ROS production. Oxidative stress arises when there is an imbalance between ROS generation and antioxidant defenses [44]. If ROS production is not adequately counteracted by enzymatic or non-enzymatic antioxidant defenses, oxidative stress events can occur, resulting in lipid and protein oxidation as well as DNA damage, which may ultimately lead to cell death and tissue injury [45]. Only a few studies have investigated the relationship between PbTx exposure and oxidative stress, most of which have focused on marine organisms [27,46,47]. In manatee plasma, a significant correlation was reported between PbTxs levels and oxidative stress markers, such as SOD and ROS, following exposure to red tide blooms [27]. In peripheral blood leukocytes from *Trachemys scripta* (loggerhead sea turtle), in vitro exposure to PbTx-3 induced upregulation of NADH:ubiquinone oxidoreductase, thioredoxin and SOD enzymes [48]. Exposure of coral larvae to PbTx resulted in increased catalase activity and elevated lipid hydroperoxidation [49]. In the present study, a clear pattern of PbTx-2 effect on ROS production in THP-1 cells was not observed. The absence of increased ROS at 4 h, followed by a decrease at 8 h, likely reflects rapid activation of the cellular antioxidant machinery rather than a lack of toxic effect. This time-dependent response suggests an adaptive phase in which the initial oxidative challenge is counteracted by upregulation of defense systems. The observed increase in *GRd* gene expression supports this, indicating active glutathione recycling to maintain redox homeostasis. Consequently, the reduction in SOD and catalase activities at 8 h may represent a secondary effect of this intensive antioxidant response, with enzymes being consumed to neutralize an early, transient oxidative burst. A decrease in SOD and catalase enzymatic activities was observed after 8 h of treatment with PbTx-2. In addition, *CAT* gene expression was also reduced following PbTx-2 exposure. Jobson et al. (2023) [50] reported a shift in the proteomic redox state of human lymphoblast cells treated with brevetoxin-2, characterized by an overall increase in protein oxidation. This effect was partially counteracted by the thiol-containing antioxidant MESNA, suggesting that its protective action may involve scavenging reactive species and restoring redox balance. *GRd* gene expression increased in response to PbTx-2, reflecting the enzyme’s role in regenerating glutathione, which is critical for xenobiotic detoxification [51]. Monocytic cells U-937 were reported to metabolize PbTx-2 via detoxification mechanisms involving the depletion of intracellular glutathione and the formation of glutathione and cysteine conjugates [30]. Therefore, the observed increase in *GRd* expression may indicate enhanced glutathione recycling to sustain its function as a detoxification mechanism.

Sas et al. (2010) [29] reported that exposure of MH-S cells to brevetoxin-2 increased secretion of interleukin IL-2, IL-4, as well as tumor necrosis factor-α, while decreasing IL-5 secretion. In the present study, THP-1 cells exposed to PbTx-2 exhibited upregulation of *IL-8* expression, which increased over time. A similar response has previously been reported in Jurkat T cells [43]. The enhanced expression of *IL-8*, a key chemokine involved in the recruitment and activation of neutrophils, suggests that PbTx-2 may promote a localized state of immune activation by stimulating cell migration.

In contrast, mRNA levels of the proinflammatory cytokines *IL-1β* and *TNF-α* were reduced at the transcriptional level. Similarly, McCall et al. (2022) [41] reported the absence of secretion of these cytokines in the same cell model. The differential regulation of *IL-8* versus *IL-1β* observed in this study is consistent with that observed in other monocytic models. McCall et al. (2022) [41] also noted a lack of classical proinflammatory cytokine secretion in THP-1 cells exposed to PbTx-2. Murrell and Gibson (2010) [43] reported that PbTx-2 interferes with proliferation signaling in lymphocytes, which may be correlated with the absence of *IL-1β* expression observed in our results. These differences likely reflect the specific voltage-gated sodium channel (VGSC) isoforms expressed in each immune cell population, leading to the prioritization of chemotactic signaling over proinflammatory activation. In Jurkat E6-1 cells exposed to PbTx-2, the expression of interleukins associated with pulmonary inflammation, such as *IL-13*, *IL-16*, *IL-8* and *IL-9*, was observed, together with an increase in neuronal apoptosis inhibitory protein (*NAIP*) expression. NAIP participates in the formation of caspase-1 activator complexes, known as inflammasomes, which lead to IL-1β secretion [43]. Collectively, these findings suggest that PbTx-2 exerts complex immunomodulatory effects, differentially regulating proinflammatory and chemotactic cytokines in a cell type-dependent manner, potentially reflecting variations in toxin sensitivity or receptor-mediated signaling pathways among immune cell populations.

In this study, the concentrations used (2.5–10 µg mL^−1^) were higher than the 3 to 30 µg L^−1^ typically measured in seawater during *Karenia brevis* blooms [17,52]. However, water concentrations primarily reflect the exposure of unicellular aquatic organisms and do not account for the internal body burden in higher vertebrates. For marine mammals and humans, exposure occurs through the consumption of contaminated prey or inhalation of toxin-containing aerosols, resulting in significant biomagnification. For example, in manatees naturally exposed to red tide events, brevetoxin levels in lung and liver tissues can reach concentrations that justify the use of µg mL^−1^ ranges in in vitro models to simulate localized cellular stress [20]. By employing human THP-1 cells, this study provides a relevant model of the systemic immune response that may occur following toxin internalization and distribution to immune-rich tissues, where local brevetoxin concentrations can far exceed those present in the surrounding environment.

These concentrations were selected to facilitate the detection of cellular events potentially affected by PbTx exposure in short-term in vitro experiments, thereby enabling subsequent exploration of the underlying mechanisms of action. Moreover, although the PbTx-2 concentrations used in this study are not typically encountered in the environment during a single exposure event, toxin circulation and bioaccumulation may lead to increased internal concentration under conditions of repeated or chronic exposure [25].

Our results suggest a time-dependent toxicological mechanism of PbTx-2 in THP-1 cells. An initial oxidative challenge, likely triggered by toxin interaction with voltage-gated sodium channels, appears to be countered by cellular antioxidant defenses. The decrease in SOD and catalase activities at 8 h, in the absence of a sustained increase in ROS levels, indicates exhaustion of the enzymatic antioxidant pool during the maintenance of redox homeostasis. Concurrently, the upregulation of *GRd* expression suggests that cells prioritize glutathione-dependent pathways to manage chemical stress. This shift in the redox state (GSH/GSSG ratio) may act as a signaling mechanism activating redox-sensitive pathways, such as NF-κB or MAPK, ultimately leading to the increased transcription of *IL-8* and *TNF-α* observed in this study [24,53,54,55]. Collectively, these findings indicate that PbTx-2 immunotoxicity involves a cascade of antioxidant depletion followed by a compensatory inflammatory response.

Although the results of this study provide valuable insights into the immunotoxic effects of PbTx-2 on THP-1 cells, several limitations should be considered. First, the use of a monocytic cell line (in vitro) represents a simplified model that does not fully capture the complex interactions among different immune cell types, or the systemic physiological responses present in vivo. Second, the concentrations of PbTx-2 employed in these experiments exceed typical environmental levels reported in water samples; however, they remain relevant for investigating acute cellular stress and potential bioaccumulation effects. Finally, as this study focused on short-term acute exposure, further research is required to evaluate the impact of chronic, low-dose exposure to PbTxs. Future studies should incorporate co-culture systems or in vivo models to better characterize the long-term consequences of these toxins on immune system integrity and the potential for cumulative tissue damage.

## 5. Conclusions

This study demonstrates that PbTx-2 at sublethal concentrations (2.8 and 5.6 µM) modulates redox and inflammatory pathways in THP-1 human monocytes. After 8 h of exposure, reduced activities of the antioxidant enzymes SOD and catalase were accompanied by increased expression of glutathione reductase and *IL-8*, together with a marked downregulation of *IL-1β*, *TNF-α*, and *CAT*. These findings indicate that PbTx-2 exerts immunomodulatory effects even under non-cytotoxic conditions, suggesting that sublethal brevetoxin exposure can alter immune cell function through oxidative stress and inflammatory response mechanisms.

The observed alterations in antioxidant defenses and proinflammatory cytokine expression support the potential for brevetoxins to contribute to immune dysfunction in exposed populations. Given the increasing frequency of harmful algal blooms associated with climate change, monitoring of PbTx levels in marine environments and seafood is essential. Further investigation of chronic low-level exposure effects on human and marine mammal immune systems is warranted to assess long-term health implications of brevetoxin bioaccumulation.

## Data Availability

The original data supporting the findings of this study are available from the corresponding author upon reasonable request.

## Abbreviations

The following abbreviations are used in this manuscript:

CBA	Cell-based assay
GPx	Glutathione peroxidase
GRd	Glutathione reductase
HAB	Harmful Algal Blooms
H_2_DCFDA	2′,7′-dichlorofluorescein diacetate
IC_50_	Half maximal inhibitory concentration
LPS	Lipopolysaccharides
MTT	3-(4,5-dimethylthiazol-2-yl)-2,5-diphenyltetrazolium bromide
PbTxs	Brevetoxines
ROS	Reactive oxygen species
STX	Saxitoxin
SOD	Superoxide dismutase enzyme
THP-1	Monocytic human leukemia cell line
TNF-α	Tumor necrosis factor
VGSC	Voltage-gated sodium channels
